# Beyond exhaustion: A multidimensional assessment of occupational stress and burnout among high school teachers

**DOI:** 10.1192/j.eurpsy.2025.1471

**Published:** 2025-08-26

**Authors:** H. Guider, F. Hadrya, Z. Boumaaize, M. A. Lafraxo, A. El Alaiki, Y. El Madhi, A. Soulaymani, A. Mokhtari, H. Hami

**Affiliations:** 1 Laboratory of Biology and Health, Faculty of Science, Ibn Tofail University, Kenitra; 2 University Hassan First of Settat, Higher Institute of Health Sciences, Health Sciences and Technologies Laboratory, Settat; 3 Higher Institute of Nursing Professions and Health Techniques, Oujda; 4 Regional Center for Education and Training Professions, Rabat, Morocco

## Abstract

**Introduction:**

In the rapidly evolving landscape of contemporary education, high school teachers are increasingly confronting significant professional stress that critically undermines their mental health and overall well-being. This pervasive issue not only affects teachers but also has broader implications for the effectiveness and sustainability of educational systems.

**Objectives:**

This study aims to elucidate the complex dynamics of occupational stress, psychosocial workplace factors, and burnout among high school teachers in Tetouan, Morocco.

**Methods:**

A cross-sectional survey was conducted among 258 high school teachers. The study utilized the Maslach Burnout Inventory (MBI) to assess burnout levels, the Job Content Questionnaire (JCQ) to evaluate psychosocial factors, and the Perceived Stress Scale (PSS-10) to measure perceived stress levels.

**Results:**

The MBI indicated that 43% of teachers experienced high emotional exhaustion, 46% reported low depersonalization, and 47% indicated low levels of personal accomplishment. On the JCQ, 55% of teachers faced high psychological demands, 57% had limited decision latitude, and 44% received insufficient social support at their workplace. There was a significant correlation between MBI emotional exhaustion and JCQ psychological demand scores (r = 0.381, p < 0.01), indicating a complex interplay between burnout dimensions and psychosocial factors in the workplace. Furthermore, the PSS-10 results exhibited a median stress score of 27, indicating a significant **variability in** stress perceptions among the participants.

**Conclusions:**

This study underscores the critical need to address burnout, psychosocial workplace factors, and perceived stress among high school teachers. The prevalent emotional exhaustion, substantial psychological demands, and varied perceived stress levels revealed by this study necessitate integrated strategies that address these complex interactions to foster a more supportive and healthy professional environment. Further research should focus on operationalizing these insights into concrete, actionable policies that enhance educational professionals’ well-being and productivity.

**Disclosure of Interest:**

None Declared

